# Assessment of health and science undergraduate students’ knowledge, attitudes, education and training related to antibiotic use and antimicrobial resistance in 27 EU/EEA universities

**DOI:** 10.1099/acmi.0.001030.v4

**Published:** 2025-10-13

**Authors:** Pak Yeung Li, Ellie L. Gilham, Sudaxshina Murdan, Orsolya Réka Süli, Rúben Viegas, Nejc Klopčič, Diane Ashiru-Oredope

**Affiliations:** 1 UCL School of Pharmacy, University College London, 29–39 Brunswick Square, Bloomsbury, London, WC1N 1AX, UK; 2 AMR and HCAI division, UK Health Security Agency, 61 Colindale Avenue, London, NW9 5EQ, UK; 3 European Medical Students’ Association (EMSA), Brussels, Belgium; 4 European Pharmaceutical Students’ Association (EPSA), Brussels, Belgium

**Keywords:** anti-infective, antimicrobial, antibiotic resistance, antibiotic stewardship, antimicrobial resistance, antimicrobial stewardship, behaviour change, European Antibiotic Awareness Day (EAAD), knowledge, students

## Abstract

**Introduction**. Antimicrobial resistance (AMR) is a complicated public health challenge. This study aimed to obtain a baseline assessment of undergraduate health and science students’ knowledge and attitudes of antibiotic use, resistance and stewardship across European countries and to evaluate education methods.

**Methods**. A 43-item cross-sectional multilingual survey of healthcare practitioners and undergraduates studying dentistry, medicine, nursing, pharmacy and science subjects was conducted by Public Health England (now UK Health Security Agency) in 2018 across 30 EU/EEA countries. Of the 43 questions developed for healthcare workers, a subset of 33 questions directly relevant to students was available for student completion.

**Results**. A total of 1,222 students from 27 EU/EEA countries participated in the survey, with 50% studying medicine (379/760). The mean score across seven knowledge questions was 6.04 out of 7 (sd, 1.14). Knowledge scores differed by the degree being studied and were higher among students in the later years of their degree programme. Knowledge was significantly higher (*P*<0.001) in those who had received training on prudent antibiotic use and infection management. Most students had not heard about AMR awareness campaigns, including European Antibiotic Awareness Day, and felt they did not have a key role in addressing AMR.

**Conclusion**. Although students demonstrated good overall knowledge of antibiotic use and AMR, many lacked awareness of their role in tackling AMR. Designing more effective targeted educational interventions for these students, such as curriculum development and interprofessional education and training, could be beneficial to support appropriate antibiotic use and efforts to tackle AMR.

## Data Summary

All data associated with this work are reported within the article and supplementary files.

## Introduction

Antimicrobial resistance (AMR) is widely recognized to be one of the most complicated public health challenges that the world has faced [1]. The contributions to AMR are multifaceted and affected by several interrelated factors, such as the nature of the micro-organism being treated with antibiotics, behavioural dynamics of healthcare professionals (HCPs), use of antimicrobials across human and animal health, poor infection prevention and control (IPC) practices, intensive farming and agriculture, societal pressures and trade and finance realities [1,2]. In 2013, it was estimated that by 2050, ~10 million deaths and an economic loss of $100 trillion would occur annually if effective interventions against AMR are not achieved [3]. To tackle rising levels of AMR, the number of antimicrobial stewardship (AMS) programmes has grown in recent years. These programmes aim to optimize antimicrobial use by targeting behavioural change to prevent the mis- and overuse of antimicrobials by the general public, as well as reduce inappropriate prescribing by HCPs [4]. In addition, HCPs have an important role in public engagement about AMR and appropriate antimicrobial consumption. The dissemination of information related to AMR [5] and to prescribing decisions to the public is potentially key to lowering the risk of resistant infections in the general public [6].

To effectively disseminate information to the public, HCPs need adequate knowledge and understanding of the subject matter. A survey of HCPs’ knowledge, attitudes and behaviours on antibiotics, antibiotic use and antibiotic resistance within 30 European countries was funded by the European Centre for Disease Prevention and Control (ECDC) and conducted by Public Health England (now UK Health Security Agency). This showed that knowledge of antibiotics and antibiotic use was high (97%), while knowledge of the development and spread of AMR was slightly lower (75%) [7].

To ensure HCPs possess the correct knowledge and attitudes about AMR and prudent antimicrobial use, education of undergraduates studying sciences and healthcare subjects is essential [8]. The Global Action Plan on AMR seeks to address this by including a focus on education and clinical training [9]. Pre-2019, research on undergraduate students’ knowledge and perception found low confidence among respondents regarding their knowledge of antibiotic use, high levels of misconception about antibiotic usage and AMR, and a desire for more education and greater understanding of guidelines on the relevant topics [10,17]. The limitations of these reports included small sample sizes and a focus on undergraduates studying only a few subjects, namely medicine [10,14], pharmacy [15,16] and dentistry [17]. Gaps in education and training related to AMR are also likely in students studying other health-related degrees, such as nursing, veterinary and science subjects, for example, biology and biomedical science. Better education on AMR and fostering appropriate behaviour regarding the prescribing, dispensing and consumption of antibiotics and integration into pre-service training is expected to be an important step in tackling AMR.

This study aimed to obtain a baseline assessment of undergraduate students’ knowledge and attitudes towards AMR and antibiotic use and prescribing in 2018 in order to inform and support future policy, as well as educational and behaviour change interventions (including campaigns) to address AMR. The survey was made available across 30 EU/EEA countries and was initially completed by HCPs [7]. The study also aimed to support the evaluation of the awareness and impact of campaigns, such as European Antibiotic Awareness Day (EAAD) and World AMR Awareness Week (WAAW) (World Antibiotics Awareness Day prior to 2023).

## Methods

### Survey setting

A cross-sectional multilingual survey of dentistry, medicine, nursing, pharmacy and science (including biology, biomedical, microbiology and nutrition) undergraduate students in 30 EU/EEA countries was conducted by Public Health England (PHE) [now UK Health Security Agency (UKHSA)] in 2018 [7]. The countries and the languages in which the survey was conducted are shown in Table S2, available in the online Supplementary Material.

### Survey design

As part of evaluating EAAD, a questionnaire for the survey was designed after previous studies indicated a gap in healthcare workers’ and healthcare students’ understanding of antibiotic use and resistance [7]. The main questionnaire and methodology have previously been reported in Ashiru-Oredope *et al*. [7]. Additional student-focused questions were included at the request of and piloted by student members of the Project Advisory Group from the European Medical Students’ Association (EMSA), European Pharmaceutical Students’ Association (EPSA) and European Dental Students Association (EDSA). These questions were translated into the 24 EU official languages, Icelandic and Norwegian. A combination of five-point Likert scale (ranging from strongly disagree to strongly agree), three-point answers (yes/no/unsure or true/false/unsure) and multiple-choice questions was used. The questions were mapped to the COM-B model of behaviour change to help identify the stages of behaviour change that should be targeted by future interventions. The COM-B model was used to develop the questionnaire as it synthesizes principles of behaviour change that are included within other behaviour change models [7]. The COM-B model specifies that behaviour is the product of an individual’s capability to perform the desired behaviour and whether they have the opportunity and motivation to carry out the behaviour [18].

### Sampling

To reach healthcare students, the survey was promoted through mailing lists by EU health student groups for medicine, pharmacy, nursing and dentistry (EMSA, EPSA and EDSA) and via social media [Facebook and X (previously known as Twitter)] using the hashtag #ECDCAntibioticSurvey.

### Data collection

The online survey questionnaire was administered using a PHE tool (surveys.phe.org.uk) in the period between 28 January 2019 to 4 March 2019. The online survey was completed independently by the participants. The background and objective of the survey were explained to respondents, and participation was voluntary and anonymous. Questions relating to HCPs’ current clinical practices were removed from the original 43-item questionnaire, with additional questions relating to teaching practices included instead, resulting in a subset of 33 questions being made available for completion by students and for analysis in the present study.

The questionnaire collected information about participants’ (i) demographics; (ii) knowledge about antibiotics, AMR and antibiotic awareness campaigns; (iii) their perception of their knowledge, their ability to deliver information to others and their role in helping control AMR; (iv) education and assessment and (v) desire for more education and training. The questions are shown in File S1.

### Data analysis

Descriptive statistics were conducted to determine respondent demographics and analyse respondents’ knowledge and understanding. For each participant, an overall knowledge score was also calculated for the seven knowledge questions by summing up the number of correct answers. Thus, the knowledge score ranged from 0 to 7, with ‘0’ showing no questions had been answered correctly and ‘7’ indicating all questions had been answered correctly. Comparisons of knowledge scores among students who had and had not received teaching on prudent antibiotic use and infection management were made using Mann–Whitney *U* tests. A *P*<0.05 significance level was used. Data analysis was conducted using Microsoft Excel and IBM SPSS Statistics Version 27.

### Ethics

This study took part as an extension to the project undertaken by Ashiru-Oredope *et al*. [7]. The study was part of an evaluation of the EU EAAD communications campaign, which commenced in 2008, and included significant input in the development and distribution of the questionnaire by EU-level student bodies. The project governance structure for this work was provided through the Project Advisory Group. The Project Advisory Group members were official representatives of all participating countries, European professional groups and organizations, including the European Pharmaceutical Students’ Association, the European Dental Students’ Association and the European Medical Students’ Association. Evaluation of the project using the NHS Research Ethics Committee (REC) assessment tool showed that ethical approval was not needed due to the focus on evaluation [19]. All respondents participated strictly in their capacity as health students and provided their informed consent by answering a consent question prior to starting the questionnaire, according to the Declaration of Helsinki. Prior to submitting their response, participants were able to withdraw from the questionnaire. However, due to responses being anonymized once a response was submitted, it could not be withdrawn. Respondents were provided with an email address they could contact if they wished to make a complaint.

## Results

### Respondents’ demographics

A total of 1,222 undergraduate students from 27 of the 30 EU/EEA countries included in the survey completed the questionnaire. Of the 30 EU/EEA countries, there were no responses from Luxembourg, Bulgaria and Iceland. Most respondents were from Italy (35%, 427/1,222), followed by the UK (9%, 111/1,222) and Austria (9%, 104/1,222) (Material S2). Most respondents were female (69%, 848/1,222) and aged 18–25 (71%, 868/1,222). Respondents were studying medicine (50%, 379/760), pharmacy (20%, 154/760), nursing (14%, 107/760), science subjects (6%, 46/760), dentistry (6%, 43/760) or other degrees, including veterinary, public health and physiotherapy (2%). Degrees being studied were unknown for 38% of respondents (462/1,222). Demographics are summarized in Material S2a, b. Except for the degree subject, the amount of missing data was low.

### Knowledge about antibiotic use and resistance

**Fig. 1.**
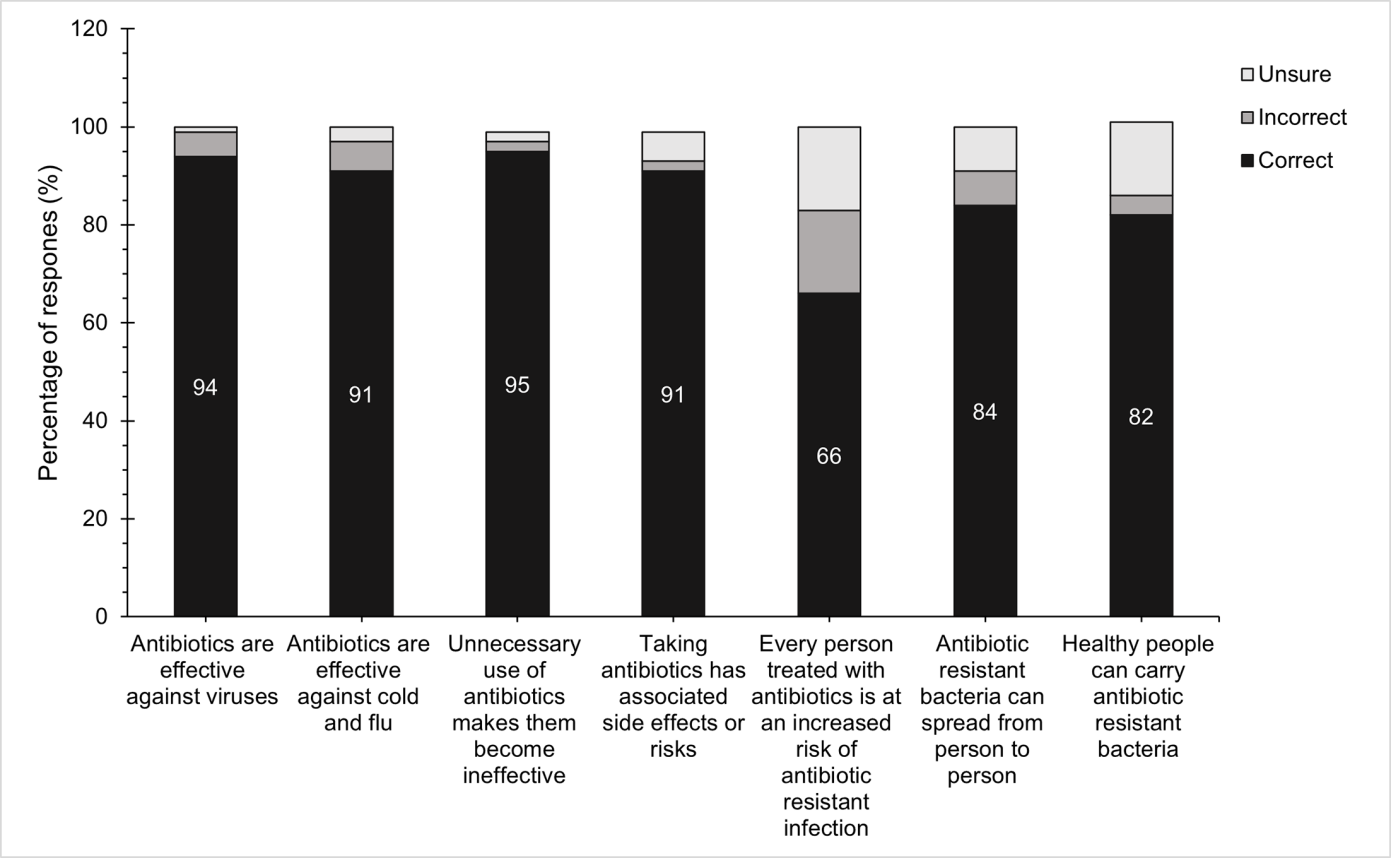
Percentage distribution of respondents answering each question correctly or incorrectly (Q7–Q13). Respondents (*n*=1,222) chose ‘True’ or ‘False’ or ‘Unsure’. ‘True’ and ‘False’ responses were converted to correct or incorrect as appropriate.

Seven questions were used to assess the respondents’ level of knowledge about antibiotic use and AMR. The percentage of respondents who answered each of these questions correctly or incorrectly is shown in Fig. 1.

The respondents' knowledge about antibiotic use was higher than their levels of knowledge about AMR (Material S3). Among the degree courses, pharmacy and medicine had the highest [51%(79/154) and 49% (187/379), respectively], while nursing and science degrees had the lowest [34%(36/105) and 33% (15/46), respectively] proportions of respondents who answered all seven knowledge questions correctly. Knowledge score increased with the amount of time spent on the degree course, with mean knowledge score increasing from 5.57 to 5.68 to 6.08 to 6.17 and to 6.30 for year 1, year 2, year 3, year 4 and year 5 or later, respectively. The proportion of respondents answering all seven knowledge questions correctly also increased from year 1 to year 3, after which no large changes were observed [year 1, 29% (17/58); year 2, 36% (39/113); year 3, 51% (55/107); year 4, 46% (98/211); year 5 or later, 52% (131/254)] (Material S4a).

Knowledge was poorest for the statement ‘Every person treated with antibiotics is at an increased risk of antibiotic-resistant infection’, with 66% (807/1,222) of respondents answering this correctly. Except for medical students (92%, 349/379) and pharmacists (90%, 139/154), knowledge was also poor regarding the statement ‘Antibiotic-resistant bacteria can spread from person to person’ (Material S4b).

### Attitudes towards antibiotic use and resistance

**Fig. 2.**
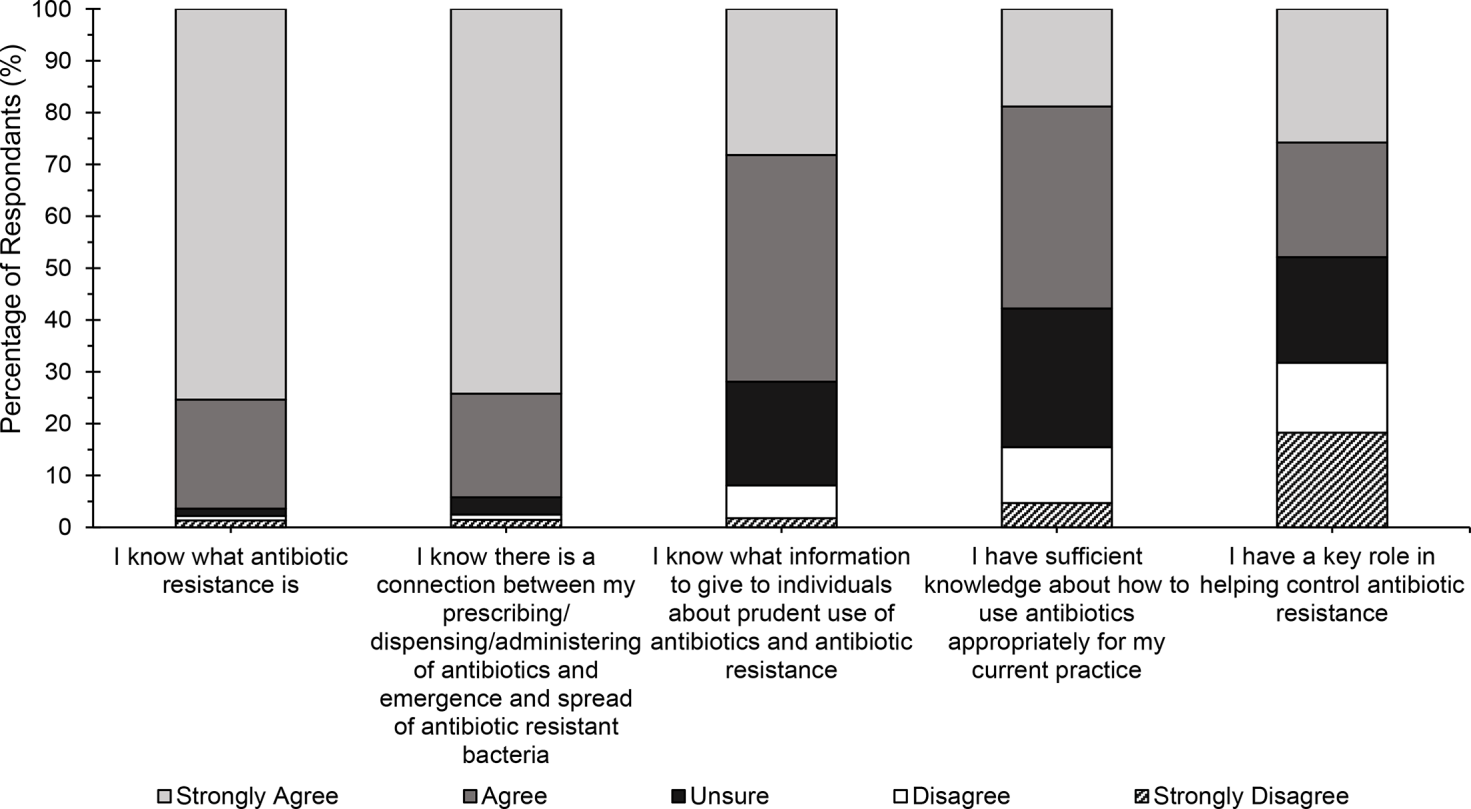
Percentage of respondents who strongly agree or agree or are undecided or disagree or strongly disagree with the statements shown above (Q14–Q18). *n*=1,040–1,218 for the different questions. Values are shown in Table S4.

Responses to questions assessing perceived capability regarding AMR and antibiotics usage are shown in Fig. 2. While most respondents strongly agreed or agreed that they know what antibiotic resistance is and that they know what information to give regarding antibiotic use and antibiotic resistance, fewer of them are sure of the connection between their antibiotic prescribing, dispensing or administering and the emergence of antibiotic resistance or of their key role in helping address antibiotic resistance.

Among the degree courses, the percentage of respondents who agreed or strongly agreed to the statements relating to perceived knowledge of antibiotic resistance (Q14) and the connection between antibiotic resistance and antibiotic usage (Q16) was similar (Table 1 and Material S5a). However, there were considerable differences for the other statements. For example, 81% of dentistry (35/43) and 79% of pharmacy students (122/154) agreed or strongly agreed that they knew what information to give about the prudent use of antibiotics and antibiotic resistance, compared to 61% of nursing (64/105) and 54% of science students (25/46). Furthermore, less than half of medical students [39% (149/379)] agreed or strongly agreed that they had sufficient knowledge about how to use antibiotics for their current practice. Only pharmacy students had a large proportion (59%, 91/154) who agreed or strongly agreed that they had a key role in helping control antibiotic resistance. Less than 50% of students from other degrees felt they had a key role in tackling antibiotic resistance.

**Table 1.** Number and percentage of respondents who strongly agree/agree with the statements shown above by degree subject

	Medicine (*n*=379)		Nursing (*n*=105)		Pharmacy (*n*=154)		Scientist (*n*=46)		Dentistry (*n*=43)	
	*n*	%	*n*	%	*n*	%	*n*	%	*n*	%
Q14: I know what antibiotic resistance is	368	97	98	93	150	97	45	98	42	98
Q16: I know there is a connection between my prescribing/dispensing/administering of antibiotics and the emergence of spread of antibiotic-resistant bacteria	351	93	92	88	142	92	41	89	40	93
Q15: I know what information to give individuals about prudent use of antibiotics and antibiotic resistance	264	70	64	61	122	79	25	54	35	81
Q17: I have sufficient knowledge about how to use antibiotics appropriately for my current practice	149	39	72	69	104	68	28	61	24	56
Q18: I have a key role in helping control antibiotic resistance	135	36	50	48	91	59	12	26	15	35

When responses were analysed by year of study, responses were found to be similar regardless of the year of study the student was in for most of the questions, except for two (Material S5b). One exception was for the statement relating to knowledge of information to give to individuals about prudent use of antibiotics and antibiotic resistance. The percentage of students who agreed or strongly agreed with this statement was 35% higher in students who had been studying for 5 or more years compared to those in their first year of study (87% vs. 52%). Another exception was for attitudes around having a key role in helping control antibiotic resistance, as there was a higher proportion of students in later years of study who agreed or strongly agreed with this statement (Y1, 44%; Y2, 53%; Y3, 45%; Y4, 65%; Y5, 63%).

### Contributors to antibiotic resistance

In terms of attitudes towards the role environmental factors have in contributing to AMR, 57% of respondents (539/945) agreed or strongly agreed that environmental factors, such as wastewater, impact on AMR. Attitudes were better for the role of antibiotic use in livestock in contributing to AMR [88% agree or strongly agree (858/975)] (Table 2). Responses were similar across degree subjects being studied by respondents.

**Table 2.** Responses to questions on the role environmental factors have in contributing to AMR

		*N*	%
Q19: The use of antibiotics to stimulate growth in farm animals is legal in the EU (*n*=1,222)	True	315	26
	False	310	25
	Unsure	597	49
Q20: Environmental factors such as wastewater in the environment (*n*=945)	Strongly disagree	40	4
	Disagree	67	7
	Unsure	299	32
	Agree	268	28
	Strongly agree	271	29
	Missing	277	–
Q21: Excessive use of antibiotics in livestock and food production (*n*=975)	Strongly disagree	20	2
	Disagree	17	2
	Unsure	80	8
	Agree	227	23
	Strongly agree	631	65
	Missing	247	–

### Awareness of antibiotic resistance

When students were asked about whether there is a good promotion of the prudent use of antibiotics and awareness of antibiotic resistance in their country, there was a clear difference among countries. Many respondents (over 60%) from Ireland, Sweden, Finland, Norway and the UK agreed with the statement. In contrast, fewer than 10% of respondents from Italy, Spain, Greece, Germany and the Netherlands agreed that there was a good promotion of the prudent use of antibiotics and awareness of antibiotic resistance in their country.

**Fig. 3.**
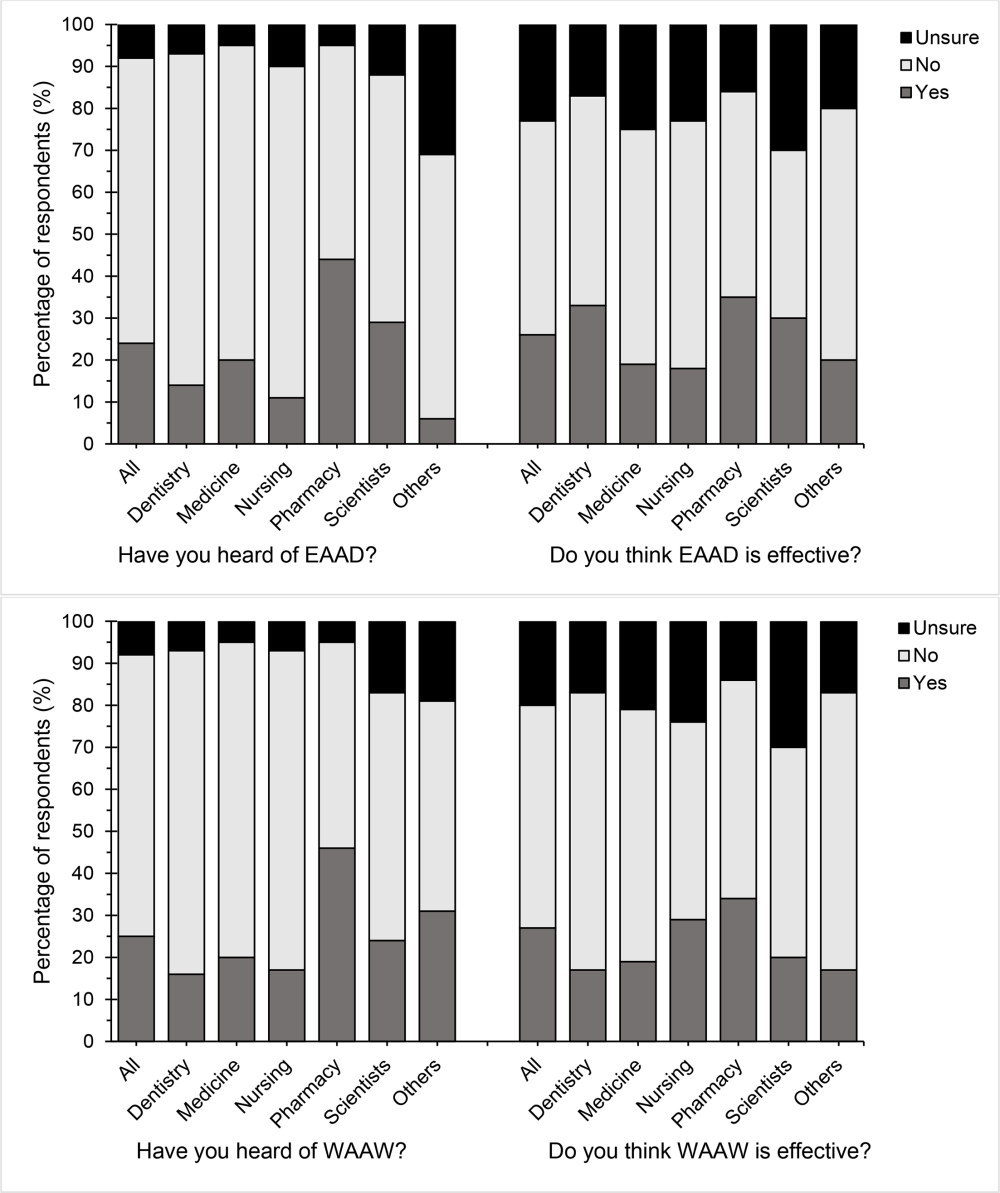
Respondents’ awareness of antibiotic awareness campaigns EAAD and WAAW (formerly World Antibiotics Awareness Day) and how effective they believe EAAD and WAAW have been in raising awareness about prudent use of antibiotics and antibiotic resistance in their country (Q26–Q27).

Most respondents (over 65%) had not heard of the EAAD or of the WAAW, and more than half of respondents were unsure how effective these two initiatives were (Fig. 3). Of all the respondents, a higher proportion of pharmacy respondents were aware of EAAD (44%) and WAAW (46%), however, even they were mostly unsure of the initiatives’ effectiveness (EAAD, 49%; WAAW, 52%) (Material S6).

**Fig. 4.**
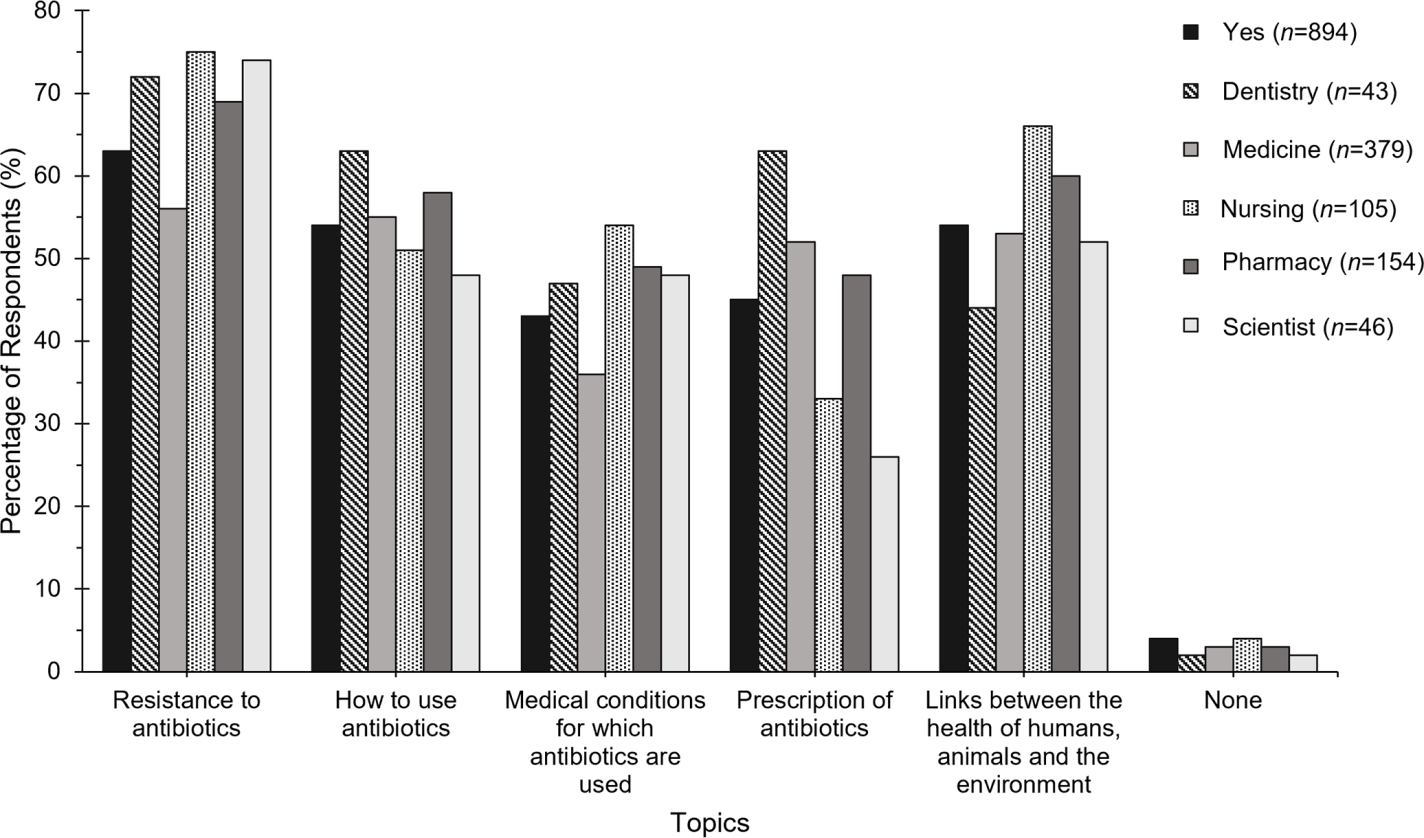
Percentage of respondents who wished to receive additional information on topics relating to AMR and prudent antibiotic use (**Q28**).

Most respondents (96%) agreed that they would like to receive further information on prudent antibiotic use and antibiotic resistance, with the most common topic being resistance to antibiotics (63%, 564/894). The topics of antibiotic use and resistance that students would like to receive more information on and are interested in are shown in Fig. 4.

### Opportunity of getting information on antibiotic prescribing

When students were asked about their ease of accessing resources linked to antibiotic use and resistance, more than half of the respondents agreed that they had easy access to guidelines (59%, 564/961) and other materials about antibiotic use and resistance (55%, 526/965).

A slightly higher proportion of nursing (67%, 67/100), pharmacy (59%, 83/141) and science (56%, 22/39) students agreed they had easy access to guidelines compared to medicine and dentistry students [(53%, 171/323) and (46%, 16/35), respectively]. The percentage of students who agreed they have easy access to materials about antibiotic use and resistance was also slightly higher among pharmacy (62%, 89/143) and nursing students (58%, 59/101).

### Training and teaching methods

More than two-thirds of the responding students reported receiving teaching and examination on prudent antibiotic use (66%, 504/758) and management of infections (69%, 521/758), with 81%(613/758) also reporting they had practical experience. Of those respondents who reported receiving teaching on prudent antibiotic use, 28% (142/504) had been studying for 4 years, and 39% (197/504) had been studying for more than 5 years. Similar proportions were also reported regarding teaching on infection management and gaining practical experience through placements or internships (Material S8).

Respondents who had received teaching and training on prudent antibiotics use had a significantly higher average knowledge score than respondents who did not receive any teaching or training (5.69±1.25 vs. 6.29±0.92; *Z*-score, −6.782; *P*<0.001) (Table 3).

**Table 3.** Mean knowledge score for respondents who had and had not received training, examination on or practical experience of antibiotic treatment, prudent antibiotic use and/or management of infection

Did you receive	Knowledge score					
	No		Yes			
	Mean	sd	Mean	sd	*Z* score	*P* value
Q32: Teaching on antibiotic treatment and prudent antibiotic use (*n*=758)	5.69	1.247	6.29	0.922	−6.782	<0.001
Q33: Teaching on management of infections (*n*=758)	5.72	1.217	6.25	0.968	−6.08	<0.001
Q34: Examinations about antibiotic treatment/prudent antibiotic use (*n*=758)	5.88	1.183	6.27	0.944	−4.641	<0.001
Q35: Examinations about management of infections (*n*=758)	5.76	1.221	6.26	9.49	−5.789	<0.001
Q37: Practical experience (internship or placement) (*n*=751)	5.73	1.226	6.17	1.026	−4.104	<0.001

*P*<0.05 was considered to be statistically significant.

Respondents’ views on the effectiveness of teaching methods on the topic of prudent antibiotic use are outlined in Material S9. Respondents thought active teaching methods, such as vignette-based clinical scenario teaching (82%, 399/487) and infectious disease clinical placements (78%, 336/433), were more useful than passive teaching methods, such as e-learning (47%, 171/368) and active learning assignments (60.4%, 255/422). Role play was thought to be the least effective teaching method, with only 41%(126/311) of students agreeing that it was useful when teaching about prudent antibiotic use.

## Discussion

### Knowledge score of respondents

Generally, students performed well when their knowledge of antibiotic use and resistance was assessed. A fairly good level of knowledge was recorded with an average of 6.04 correct answers out of seven questions and 44% of respondents answered all seven questions correctly. As expected, the students’ knowledge score was slightly lower than that of HCPs from the EU/EEA countries who answered an average of 6.35 out of seven questions correctly and 58% of whom answered all questions correctly [7]. Knowledge tended to be lower for questions relating to the increased risk of resistant infection following treatment with antibiotics. This suggests it is important to build education into undergraduate training programmes, as this appears to translate into similar levels of knowledge for qualified HCPs [20].

Knowledge scores improved from the first year to the fifth year of study. This may be due to education sessions on antibiotic use and resistance generally being delivered in later years of study and/or students consolidating their knowledge before graduation in order to prepare for progressing into real-life practice. However, it is worth noting that not all courses will extend over a 5-year period, especially science-related courses. In addition, not all students studying health and science-related courses in all countries will become future prescribers. While health professionals (especially doctors, dentists, nurses and pharmacists) will have a role in antibiotics prescribing, administration or dispensing, not all may be involved in prescribing of antibiotics. This means some subjects may not include as much information relating to prescribing antibiotics. As a result, this may impact student responses, especially those relating to the connection between prescribing and AMR.

Pharmacy and medicine undergraduates were most likely to answer all knowledge questions correctly, as well as having the highest mean knowledge score. This may be explained by the greater number of teaching hours dedicated to AMS in medical school and pharmacy courses compared to nursing and dentistry courses [21]. When assessing HCPs’ knowledge of AMR and antibiotic prescribing, these professions also showed the highest levels of knowledge, with 68% of medical doctors and 59% of pharmacists answering all knowledge questions correctly. This suggests that knowledge gained during undergraduate studies may transfer into clinical practice [7]. However, scores for students studying medicine and pharmacy were lower when compared to qualified doctors and pharmacists. This suggests that dedicating more time and effort to AMS teaching within degree programmes for other healthcare workers may have a positive impact on students’ knowledge of antibiotic resistance and prudent antibiotic use and prevent students from commencing their careers with a lower level of knowledge, which may have a subsequent impact on AMR.

Furthermore, all the students’ knowledge regarding environmental contributors to AMR was relatively low. This is of concern due to the increasing focus on the use of a One Health approach to tackle AMR as outlined within government strategies on AMR [22].

### Perceived capacity and motivation of respondents on antibiotic use and resistance

Most respondents agreed that they know what antibiotic resistance is and have an understanding of the connection between administering antibiotics and the spread of antibiotic resistance, suggesting a high level of capability among students to implement behaviours that would positively impact AMR. This suggests that although students may not perceive they have formal teaching sessions, learning is taking place and students do feel that they have an understanding of the topic area. However, this study did not explore differences between teaching and learning and did not determine whether students were accessing any learning materials from other sources. Therefore, future work may wish to further explore these differences as well as the resources that are available to students outside of the university setting.

Less than half of the undergraduates (48%) agreed they have a role in helping control antibiotic resistance, suggesting they do not feel they will have opportunities in their future careers to implement behaviours that promote appropriate antibiotic use. Furthermore, fewer than half of medical students felt they had sufficient knowledge on the use of antibiotics for their current practice. This may reflect that undergraduate students lack confidence in their levels of knowledge regarding AMR and appropriate antibiotic use or underestimate their role in tackling antibiotic resistance. This may be due to insufficient teaching on antibiotic use or a lack of opportunity to implement the content medical students have been taught. The high proportion of respondents feeling they do not have a key role in tackling AMR may be due to respondents thinking of themselves as students in the present and the little opportunity they have currently to affect antibiotic resistance, rather than thinking of the future opportunities they would have when they become practitioners or scientists. However, behaviours that do not promote AMS may develop if students continue to underestimate their role in tackling AMR after moving into professional practice.

### Awareness of antibiotic stewardship and public awareness campaigns

Following the selection of AMR by the World Health Organisation (WHO) as the main theme of World Health Day in 2011, multiple AMS projects which aim to address the problem of AMR have been developed and promoted nationally and globally, including EAAD commenced in 2008 [23], Antibiotic Guardian launched in 2014 [24], eBug [25] and WAAW commenced in 2015 [26].

Evidence suggests that AMS campaigns may play a key role in reducing antibiotic consumption, with public health campaigns shown to have reduced the mean level of overall antibiotic consumption in Europe between 1997 and 2007 by 6.5–28.3% [27]. However, only a quarter of the present study’s respondents had heard of EAAD (24%) or WAAW (25%), with the highest levels of awareness seen among pharmacy students (EAAD, 44%; WAAW, 46%) and the lowest among nursing students (EAAD, 11%; WAAW, 17%). Predictably, awareness of EAAD and WAAW was higher among undergraduates who had been studying for five or more years compared to those who were in their first year of their degree. Further analysis showed that half of the respondents were undecided about the effectiveness of EAAD and WAAW. However, this finding was likely to have been influenced by the low levels of student awareness of these campaigns. Awareness campaigns that involve HCP education and use interaction between HCP and the public tend to see improvements in the study’s primary outcome measure [5]. Therefore, increasing HCP and students’ awareness of these initiatives is likely to be beneficial. However, most studies evaluating AMR awareness campaigns have used observational study designs, making it difficult to determine causality and thus the true effectiveness of the campaign [5]. As a result, future work is needed to determine campaign effectiveness with more certainty and ensure that findings are disseminated more widely; this may, in turn, increase HCP and students’ motivation to engage with campaigns.

Standardizing teaching on AMS may also help improve awareness of AMS initiatives and is recommended for all healthcare students [21]. Ensuring consistent content on knowledge and practice is likely to promote professional development, ensure quality assurance requirements are met and allow students to clearly define their roles and responsibilities. Standardization of teaching may also prevent knowledge gaps and reduce the wide variability among students from different degree courses in awareness of antibiotic awareness campaigns identified in this study. It may also be beneficial to embed the concepts of AMS earlier in preclinical sections of degree courses.

### Educational interventions on antibiotic use and resistance

In this study, we have made a comparison between knowledge score and engagement in different educational activities such as teaching, examination and work placement (Table 3). The mean knowledge score was significantly higher (*P*<0.001) among students who had received teaching and examination and/or had practical experience on the prudent antibiotic use and management of infections. On assessing the usefulness of teaching methods, this study showed that students perceived more active teaching methods, such as vignette-based clinical scenario teaching, which involves students engaging in hypothetical patient scenarios to develop their clinical decision-making skills [28] and clinical placements to be more effective than passive teaching methods, such as classical formal lectures and small group teaching. This finding is supported by the literature [29], for example, Abdel Meguid *et al*. examined students’ perceptions of teaching methods and found that the use of interactive teaching methods resulted in a significant increase in students’ motivation and engagement in learning [30]. Despite this, traditional passive teaching methods, such as lectures, are primarily applied across European universities to instruct the learning on prudent antibiotic use [31]. This may be due to the level of comfort teaching staff and students have with more traditional teaching methods, as well as the additional time and resource requirements necessary to successfully implement more active teaching methods [29,32]. Therefore, it may be beneficial for future work to evaluate teaching methods to determine which methods should be promoted within universities and implemented by educators to facilitate learning and improve knowledge of AMR and antibiotic use. It may also be helpful if a repository of resources, e.g. clinical vignettes across common infections and entrustable professional activities during clinical placements, is made available which can be adapted by those leading on the education of health students in particular [33,34]. The use of qualitative research techniques would also allow students’ perspectives on teaching and training on AMR, including the teaching styles that they find most beneficial, to be explored further.

One key challenge to address is how sufficient time can be spent educating on important principles of AMR, antimicrobial use and AMS and how this could be included consistently in degree curricula across nations, especially given the recurring issue of insufficient time to add additional elements to many degree curricula due to rigid, crowded and constrained curriculum policies within EU healthcare courses [31]. Furthermore, the perception that healthcare students have regarding teaching on any topic also creates challenges, as students may not perceive that a topic has sufficient teaching time attached to it. One potential solution could be to include information on AMR and AMS within modules that are taught on related subjects, for example, through vertical integration [35]. Although vertical integration is defined in the context of health education as the ‘integration between the clinical and basic science parts throughout the programme’, principles required to manage infections, including IPC and AMS, can be integrated across the education of undergraduate health students. This would prevent the need to include additional modules on these areas, thus reducing teaching burden on university staff. Furthermore, utilizing resources that have already been developed specifically to be applicable across multiple health contexts and nations, such as WHO AMS education and training resources [36] and those available through the Flemming Fund [37] or others, may reduce the burden on teaching staff relating to the need to develop module content relating to this topic area. Outlining the importance of education within countries’ National Action Plans (NAP), such as the UK NAP, may also aid in the progression towards standardized inclusion of information on AMR within health science degrees.

### Strengths and limitations

This study has several strengths. Firstly, to the best of our knowledge, at the time of the study in 2018, this was the first study to survey multi-disciplinary health-related and science-related students at the same time. Furthermore, the sample size was higher than previous studies on similar topics, with a difference of 15–148% between the sample in the present study compared to previous studies [10,13, 38, 39]. The present study was also conducted across 27 European countries and was multilingual. This ensured we were able to collect data from individuals who may not read English as their first language, which may impact understanding of the questions being asked, thus improving the representativeness and generalizability of the findings.

Nevertheless, the study also has some limitations, which mainly relate to the use of self-administered questionnaires, whereby survey questions were not asked of the participant by a member of the research team. Social desirability bias may have led to respondents using online resources when answering knowledge-based survey questions to appear more knowledgeable on a subject, leading to potential over-reporting of the knowledge score and thus an overestimation of the levels of knowledge students are gaining through their university degrees. Self-administered questionnaires and the use of convenience sampling are also associated with bias, as students who are engaged and interested in the topic of AMR are more likely to complete the questionnaire.

Secondly, the sample size of respondents from different countries varied widely, ranging from 3 to 427, meaning comparisons between countries could not be conducted. While robust sample sizes were collected within some countries and degree subjects, it is unclear why the questionnaire received low response rates from others, including Germany, Denmark, the Netherlands and Ireland, as well as from degree subjects such as dentistry. Future work could focus on areas with low response rates to improve our understanding of AMR knowledge among students studying within those countries, as well as among students studying specific health courses.

Finally, further information on degree courses was not obtained; therefore, there may be significant variation in degree structure, content and teaching hours provided by universities, all of which may influence questionnaire responses. This highlights the potential value and importance of introducing more standardized curricula and examination processes within higher education settings, although this would be challenging to enforce. Furthermore, some of the comparisons between degree subjects should be treated with caution, as students studying some courses may not have received any clinical skills training, as they are unlikely to move into a patient-facing role following completion of their course.

## Conclusion

Our exploratory evaluation study identified that more than half of the students who responded to the survey were not aware of antibiotic awareness campaigns such as EAAD and WAAW, although awareness tended to increase with the year of study. The study also highlighted that students generally have a higher level of knowledge on the topic of antimicrobial use compared to AMR. Among degree courses, pharmacy and medicine undergraduates tended to have the highest levels of knowledge. As expected, the knowledge score increased gradually from year 1 to year 5 or later.

As expected, students’ knowledge was significantly higher when they were taught, examined or had undertaken professional placements on prudent antibiotic use and infection management. Therefore, designing targeted educational interventions, such as standardization of curricula and interprofessional development, may make future HCPs better prepared to promote prudent use of antibiotics, among other factors that contribute towards AMR. Countries could also utilize the survey results as a baseline for evaluation of the effectiveness of interventions, such as providing antibiotic resources and guidelines to improve students’ knowledge on AMR and prudent antibiotic use .

## Supplementary material

10.1099/acmi.0.001030.v4 Uncited Supplementary Material 1.

## Abbreviations

AMR: antimicrobial resistance
AMS: antimicrobial stewardship
EAAD: European Antibiotic Awareness Day
ECDC: European Centre for Disease Prevention and Control
EDSA: European Dental Students Association
EEA: European Economic Area
EMSA: European Medical Students’ Association
EPSA: European Pharmaceutical Students’ Association
EU: European Union
HCPs: healthcare professionals
NAP: National Action Plans
NHS: National Health Service
PHE: Public Health England
REC: Research Ethics Committees
WAAW: World Antimicrobial Resistance Awareness Week
WHO: World Health Organisation
