# Supplementary material for: Assessment of health and science undergraduate students’ knowledge, attitudes, education and training related to antibiotic use and antimicrobial resistance in 27 EU/EEA universities
Source: Access Microbiol. 2025 Oct 13;7(10):001030.v4. doi: 10.1099/acmi.0.001030.v4 (PMC12518050; doi:10.1099/acmi.0.001030.v4)
Supplement: Uncited Supplementary Material 1. [file acmi-7-01030-s001.pdf]

Supplementary Material 1: Survey Questions

Part 1.Demographic

Q 1	In what country are you currently studying?<br>Multiple choice question<br>Select from one of the following :<br><br>Austria ; Belgium ; Croatia ; Cyprus ; Czech Republic ; Denmark ; Estonia ; Finland ; France ; Germany ; Greece ; Hungary ; Ireland ; Italy ; Latvia ; Lithuania ; Malta ; Netherlands ; Norway ; Others ; Poland ; Portugal ; Romania ; Slovakia ; Slovenia ; Spain ; Sweden ; United Kingdom
Q 2	What career are you currently studying for?<br>Multiple choice question<br>Select from one of the following :<br><br>Medicine ; Nursing ; Pharmacy ; Dentistry ; Scientist ; Other
Q 3	What year of your studies are you in?<br>Multiple choice question<br>Select from one of the following :<br><br>1st year ; 2nd year ; 3rd year ; 4th year ; 5th year or later
Q 4	What is your age?<br>Multiple choice question<br>Select from one of the following :<br><br>18-25 years ; 26-35 years ; 36-45 years ; 46-55 years ; 56-65 years ; >66 years ; I prefer not to say
Q 5	What gender do you most identify with?<br>Multiple choice question<br>Select from one of the following :<br><br>Male ; Female ; I prefer not to say
Q 6	Which of the following social media networks do you mainly use for professional activities?<br>Multiple choice question<br>Select from no more than 2 of the following :<br><br>Twitter ; Facebook ; LinkedIn ; Google+ ; YouTube ; Instagram ; I do not use social media ; other

Part 2. Knowledge

Please answer whether you believe these statements are true or false.
Multiple choice questions
Select from one of the following :

True ; False ; Unsure

Q 7 Antibiotics are effective against viruses

Q 8 Antibiotics are effective against cold and flu

Q 9 Unnecessary use of antibiotics makes them become ineffective

Q 10 Taking antibiotics has associated side effects or risks such as diarrhoea, colitis, allergy

Q 11 Every person treated with antibiotics is at an increased risk of antibiotic resistant infection

Q 12 Antibiotic resistant bacteria can spread from person to person

Q 13 Healthy people can carry antibiotic resistant bacteria

Part 3. Perceived capacity and motivation

To what extent do you agree or disagree with the following statements?
Likert scale questions
Select from one of the following :

Strongly Disagree ; Disagree ; Undecided ; Agree ; Strongly Agree ; I do not understand the question ; N/A

Q 14	I know what antibiotic resistance is
Q 15	I know what information to give to individuals about prudent use of antibiotics and antibiotic resistance
Q 16	I know there is a connection between my prescribing OR dispensing OR administering of antibiotics and emergence and spread of antibiotic resistant bacteria
Q17	I have sufficient knowledge about how to use antibiotics appropriately for my current practice
Q 18	I have a key role in helping control antibiotic resistance

Part 4. Contributors to antibiotic resistance

To what extent do you agree or disagree that the following environmental and animal health factors are important in contributing to antibiotic resistance in bacteria from humans?
Likert scale questions
Select from one of the following :

Strongly Disagree ; Disagree ; Undecided ; Agree ; Strongly Agree ; I do not understand the question

Q19 The use of antibiotics to stimulate growth in farm animals is legal in the EU

Q 20 Environmental factors such as waste water in the environment

Q 21 Excessive use of antibiotics in livestock and food production

Part 5. Awareness of antibiotic resistance

Q 22 At what level do you think it is most effective to tackle resistance to antibiotics?
Multiple choice questions
Select from no more than 2 of the following :

Individual level (public) ; Individual level (prescribers) ; Individual level (all healthcare workers) ; Environmental / Animal Health ; Regional/ National Level ; EU/ Global ; Action at all levels needed ; I do not know

Q 23 What initiatives are you aware of in your country which focus on antibiotic awareness and resistance?
Multiple choice questions
Select all that apply of the following :

TV or Radio advertising for the public ; Toolkits and resources for healthcare workers ; National or regional guidelines on management of infections ; Awareness raising from professional organisations ; Conference/ Events focused on tackling antibiotic awareness ; Newspaper (national) articles on antibiotic resistance ; National campaign ; World Antibiotic Awareness Week/ European Antibiotic Awareness Day ; I am not aware of any initiatives ; Other

To what extent do you agree or disagree with the following statements regarding the national initiatives about prudent use of antibiotics in your country?
Likert scale questions
Select from one of the following :

Strongly Disagree ; Disagree ; Undecided ; Agree ; Strongly Agree ; I do not understand the question ; Not applicable

Q 24 There has been good promotion of prudent use of antibiotics and antibiotic resistance in my country

Q 25 I believe the national campaign has been effective in reducing unnecessary antibiotic use and controlling antibiotic resistance

---

Q 26 Have you heard of European Antibiotic Awareness Day (EAAD) or World Antibiotic Awareness Week (WAAW)?
Multiple choice questions
Select from one of the following :

Yes ; No ; Unsure

Q 27 How effective do you believe EAAD and WAAW have been in raising awareness about prudent use of antibiotics and antibiotic resistance in your country?
Likert scale questions
Select from one of the following :

Strongly Disagree ; Disagree ; Undecided ; Agree ; Strongly Agree ; I do not understand the question

---

Q 28 On which topics would you like to receive more information?
Multiple choice questions
Select from all that apply of the following :

Resistance to antibiotics ; How to use antibiotics ; Medical conditions for which antibiotics are used ; Prescription of antibiotics ; Links between the health of humans, animals and the environment ; None ; Other

Part 6. Opportunity of getting antibiotic information

To what extent do you agree or disagree with the following statements?
Likert scale questions
Select from one of the following :

Strongly Disagree ; Disagree ; Undecided ; Agree ; Strongly Agree ; I do not understand the question ; Not applicable

Q 29 I have easy access to guidelines I need on managing infections

Q 30 I have easy access to the materials I need to give advice on prudent antibiotic use and antibiotic resistance

Q 31 I have good opportunities to provide advice on prudent antibiotic use to individuals

Part 7. Training and teaching method

Q 32 Have you had any teaching about antibiotic treatment and prudent antibiotic use during your undergraduate degree
Multiple choice questions
Select from one of the following :

Yes ; No ; Unsure

Q33 Have you had any teaching on the management of infections during your undergraduate degree?
Multiple choice questions
Select from one of the following :

Yes ; No ; Unsure

Q 34 Have any of your examinations included questions about antibiotic treatment or prudent use of antibiotics?
Multiple choice questions
Select from one of the following :

Yes ; No ; Unsure

Q35 Have any of your examinations included questions about the management of infections? Multiple choice questions
Select from one of the following :

Yes ; No ; Unsure

Q 36 Which of the following methods of teaching have been used to teach you about prudent use of antibiotics/antibiotic treatment and how useful would you rate them?

Teaching methods :

- Lectures (with >15 people)
- Small group teaching (with <15 people)
- Discussions of clinical cases and vignettes
- Active learning assignments (e.g article reading, group work, preparing an oral presentation)
- E-learning
- Role play or communication skills sessions dealing with patients demanding antibiotic training
- Infectious diseases clinical placement (i.e. clinical rotation or training in infectious diseases, involving patients)
- Microbiology clinical placement
- Peer or near peer-teaching (i.e. teaching led by other students or recently qualified doctors)

<u>Likert scale questions</u>
Select from one of the following :

Strongly Disagree ; Disagree ; Undecided ; Agree ; Strongly Agree ; I do not understand the question ; Not applicable

---

Q 37 Have you had any practical experience of your future profession (internship or placement)
<u>Multiple choice questions</u>
Select from one of the following :

Yes ; No ; I do not understand the question

Supplementary Material 2a Country in which the respondent was studying

**Country in which respondent was studying**	**Frequency (N)**	**Percentage (%)**	**Country in which respondent was studying**	**Frequency (N)**	**Percentage (%)**
Italy	427	35	Spain	16	1
United Kingdom	111	9	Belgium	13	1
Austria	104	9	Czech Republic	13	1
Finland	76	6	Germany	12	1
Norway	69	6	Denmark	9	1
France	59	5	Estonia	9	1
Hungary	56	5	Croatia	9	1
Poland	42	3	Cyprus	8	1
Slovakia	37	3	Ireland	7	1
Slovenia	33	3	Latvia	5	0.4
Greece	31	3	Malta	5	0.4
Sweden	24	2	Netherlands	5	0.4
Romania	18	2	Lithuania	3	0.2
Portugal	17	1	Others	4	0.3
			Total	1222	100

Supplementary Material 2b. Respondent demographics including career studying for, number of years studying for, age and sex

		**n**	**%**
**Career currently studying for**	Medicine	379	50
	Pharmacy	154	20
	Nursing	105	14
	Scientist	46	6
	Dentistry	43	6
	Others	16	2
	Unknown	17	2
	Missing	462	
**Number of years studying for**	1	59	8
	2	113	15
	3	108	14
	4	211	28

	5 or later	254	34
	Unknown	9	1
	Missing	468	
**Age**	18-25	868	71
	26-35	261	21
	36-45	52	4
	46-55	28	2
	56-65	8	1
	>66	3	0.2
	Prefer not to say	2	0.2
**Sex**	Female	848	69
	Male	350	29
	Prefer not to say	24	2
	Total	1222	100

Supplementary Material 3. Percentage distribution of respondents answering correctly or incorrectly on the questions assessing knowledge of antibiotic use and resistance (n=1,222)

	Correct Answer	Correct		Incorrect		Unsure	
		n	%	n	%	n	%
Antibiotics are effective against viruses	FALSE	1150	94	55	5	17	1
Antibiotics are effective against cold and flu	FALSE	1117	91	70	6	35	3
Unnecessary use of antibiotics makes them become ineffective	TRUE	1166	95	27	2	29	2
Taking antibiotics has associated side effects or risks such as diarrhoea, colitis, allergies	TRUE	1116	91	28	2	78	6
Every person treated with antibiotics is at an increased risk of antibiotic resistant infection	TRUE	807	66	203	17	212	17
Antibiotic resistant bacteria can spread from person to person	TRUE	1025	84	91	7	106	9
Healthy people can carry antibiotic resistant bacteria	TRUE	998	82	45	4	179	15

Supplementary Material 4a. Number and percentage of correctly answered knowledge questions by year of study and degree course

		Number of correct responses													
		1		2		3		4		5		6		7	
		n	%	n	%	n	%	n	%	n	%	n	%	n	%
Year of Study	Year 1	0	0.0	0	0.0	4	6.9	7	12.1	16	27.6	14	24.1	17	29.3
	Year 2	1	0.9	3	2.7	6	5.3	7	6.2	26	23.0	31	27.4	39	34.5
	Year 3	0	0.0	0	0.0	5	4.7	8	7.5	15	14.0	24	22.4	55	51.4
	Year 4	0	0.0	0	0.0	3	1.4	10	4.7	34	16.1	66	31.3	98	46.4
	Year 5+	0	0.0	0	0.0	2	0.8	8	3.1	34	13.4	79	31.1	131	51.6
Degree course	Dentistry	0	0.0	0	0.0	4	9.3	4	9.3	8	18.6	10	23.3	17	39.5
	Medicine	1	0.3	2	0.5	6	1.6	17	4.5	55	14.5	111	29.3	187	49.3
	Nursing	0	0.0	1	1.0	2	1.9	8	7.6	24	22.9	34	32.4	36	34.3
	Other	0	0.0	0	0.0	0	0.0	1	6.3	4	25.0	2	12.5	9	56.3
	Pharmacy	0	0.0	0	0.0	2	1.3	5	3.2	22	14.3	46	29.9	79	51.3
	Scientific	0	0.0	0	0.0	3	6.5	4	8.7	8	17.4	16	34.8	15	32.6
	Unknown	0	0.0	0	0.0	3	17.6	1	5.9	6	35.3	2	11.8	5	29.4

Supplementary Material 4b. Percentage distribution of respondents answering correctly or incorrectly on the questions assessing knowledge of antibiotic use and resistance by degree course being studied

		Dentistry (n=42)		Medicine (n=379)		Nursing (n=105)		Others (n=25)		Pharmacy (n=154)		Scientist (n=55)		Unknown (n=462)	
		n	%	n	%	n	%	n	%	n	%	n	%	n	%
Antibiotics are effective against viruses	Correct	36	85.7	370	97.6	93	88.6	24	96.0	148	96.1	52	94.5	427	92.4
	Incorrect	5	11.9	7	1.8	11	10.5	1	4.0	5	3.2	3	5.5	23	5.0
	Unsure	1	2.4	2	0.5	1	1.0	0	0.0	1	0.6	0	0.0	12	2.6
Antibiotics are effective against colds and flu	Correct	37	88.1	359	94.7	92	87.6	20	80.0	148	96.1	45	81.8	416	90.0
	Incorrect	2	4.8	14	3.7	9	8.6	2	8.0	4	2.6	6	10.9	33	7.1
	Unsure	3	7.1	6	1.6	4	3.8	3	12.0	2	1.3	4	7.3	13	2.8
Unnecessary use of antibiotics makes them become ineffective	Correct	41	97.6	357	94.2	101	96.2	24	96.0	151	98.1	53	96.4	439	95.0
	Incorrect	0	0.0	9	2.4	2	1.9	1	4.0	3	1.9	1	1.8	11	2.4
	Unsure	1	2.4	13	3.4	2	1.9	0	0.0		0.0	1	1.8	12	2.6
Taking antibiotics has associated side effects or risks such as diarrhoea, colitis, allergies	Correct	39	92.9	347	91.6	99	94.3	25	100.0	138	89.6	47	85.5	421	91.1
	Incorrect	2	4.8	9	2.4	1	1.0	0	0.0	2	1.3	2	3.6	12	2.6
	Unsure	1	2.4	23	6.1	5	4.8	0	0.0	14	9.1	6	10.9	29	6.3
Every person treated with antibiotics is at an increased risk of antibiotic resistant infection	Correct	26	61.9	250	66.0	74	70.5	14	56.0	108	70.1	35	63.6	300	64.9
	Incorrect	9	21.4	73	19.3	12	11.4	3	12.0	22	14.3	10	18.2	74	16.0
	Unsure	7	16.7	56	14.8	19	18.1	8	32.0	24	15.6	10	18.2	88	19.0
Antibiotic resistant bacteria can spread from person to person	Correct	31	73.8	349	92.1	76	72.4	16	64.0	139	90.3	42	76.4	372	80.5
	Incorrect	7	16.7	14	3.7	16	15.2	5	20.0	4	2.6	7	12.7	38	8.2
	Unsure	4	9.5	16	4.2	13	12.4	4	16.0	11	7.1	6	10.9	52	11.3
Healthy people can carry antibiotic resistant bacteria	Correct	34	81.0	309	81.5	81	77.1	17	68.0	133	86.4	44	80.0	380	82.3
	Incorrect	2	4.8	15	4.0	4	3.8	2	8.0	4	2.6	0	0.0	18	3.9
	Unsure	6	14.3	55	14.5	20	19.0	6	24.0	17	11.0	11	20.0	64	13.9

Supplementary Material 5a. Students' responses to questions assessing the perceived capability of respondents on AMR and prescribing by degree course, n = 1222

		Strongly Disagree		Disagree		Unsure		Agree		Strongly Agree		Missing
		n	%	n	%	n	%	n	%	n	%	
All	I know what antibiotic resistance is	16	1	11	1	17	1	256	21	918	75	4
	I know there is a connection between my prescribing/dispensing/administering of antibiotics and emergence and spread of resistant bacteria	17	1	12	1	39	3	234	20	870	74	50
	I know what information to give to individuals about prudent use of antibiotics and AMR	21	2	75	6	238	20	520	44	335	28	33
	I have sufficient knowledge about how to use antibiotics appropriately for my current practice	53	5	122	11	303	27	441	39	213	19	90
	I have a key role in helping control AMR	190	18	140	13	212	20	230	22	268	26	182
Dentistry	I know what antibiotic resistance is	0	0	0	0	1	2	6	14	36	84	0
	I know there is a connection between my prescribing/dispensing/administering of antibiotics and emergence and spread of resistant bacteria	1	2	0	0	2	5	3	7	37	86	0
	I know what information to give to individuals about prudent use of antibiotics and AMR	0	0	2	5	5	12	19	46	16	39	1
	I have sufficient knowledge about how to use antibiotics appropriately for my current practice	1	3	6	17	8	23	19	54	5	14	4
	I have a key role in helping control AMR	3	10	4	14	14	48	7	24	8	28	7
Medicine	I know what antibiotic resistance is	2	1	3	1	6	2	75	20	293	77	0
	I know there is a connection between my prescribing/dispensing/administering of antibiotics and emergence and spread of resistant bacteria	3	1	3	1	9	3	47	13	304	86	13
	I know what information to give to individuals about prudent use of antibiotics and AMR	6	2	24	7	79	22	181	49	83	23	6
	I have sufficient knowledge about how to use antibiotics appropriately for my current practice	23	7	47	15	129	41	117	37	32	10	31
	I have a key role in helping control AMR	80	32	48	19	53	21	61	24	74	29	63

Nursing	I know what antibiotic resistance is	5	5	1	1	1	1	29	28	69	66	0
	I know there is a connection between my prescribing/dispensing/administering of antibiotics and emergence and spread of resistant bacteria	4	4	1	1	4	4	32	33	60	62	4
	I know what information to give to individuals about prudent use of antibiotics and AMR	4	4	7	7	26	27	40	41	24	25	4
	I have sufficient knowledge about how to use antibiotics appropriately for my current practice	1	1	10	11	16	17	44	47	28	30	6
	I have a key role in helping control AMR	7	10	10	14	22	30	34	47	16	22	16
Other	I know what antibiotic resistance is	0	0	2	13	0	0	3	19	11	69	0
	I know there is a connection between my prescribing/dispensing/administering of antibiotics and emergence and spread of resistant bacteria	0	0	2	14	0	0	2	14	11	79	1
	I know what information to give to individuals about prudent use of antibiotics and AMR	0	0	3	21	0	0	8	57	4	29	1
	I have sufficient knowledge about how to use antibiotics appropriately for my current practice	0	0	3	30	1	10	6	60	3	30	3
	I have a key role in helping control AMR	0	0	3	38	4	50	2	25	3	38	4
Pharmacy	I know what antibiotic resistance is	3	2	0	0	1	1	25	16	125	81	0
	I know there is a connection between my prescribing/dispensing/administering of antibiotics and emergence and spread of resistant bacteria	2	1	2	1	4	3	32	22	110	75	4
	I know what information to give to individuals about prudent use of antibiotics and AMR	2	1	4	3	22	15	74	51	48	33	4
	I have sufficient knowledge about how to use antibiotics appropriately for my current practice	2	1	2	1	38	28	77	56	27	20	8
	I have a key role in helping control AMR	11	8	17	13	24	18	31	23	60	45	11
Scientific	I know what antibiotic resistance is	1	2	0	0	0	0	4	9	41	89	0
	I know there is a connection between my prescribing/dispensing/administering of antibiotics and emergence and spread of resistant bacteria	1	3	0	0	1	3	8	20	33	83	3

	I know what information to give to individuals about prudent use of antibiotics and AMR	1	2	4	10	14	33	11	26	14	33	2
	I have sufficient knowledge about how to use antibiotics appropriately for my current practice	3	9	2	6	7	21	18	53	10	29	6
	I have a key role in helping control AMR	10	33	7	23	9	30	6	20	6	20	8

Supplementary Material 5b. Students responses to questions assessing the perceived capability of respondents on AMR and prescribing by year of study

		Strongly Disagree		Disagree		Unsure		Agree		Strongly Agree		
Year of study		n	%	n	%	n	%	n	%	n	%	Missing
1 year	I know what antibiotic resistance is	0	0	4	7	2	4	16	30	36	67	4
	I know there is a connection between my prescribing/dispensing/administering of antibiotics and emergence and spread of resistant bacteria	0	0	3	6	4	7	16	30	31	57	4
	I know what information to give to individuals about prudent use of antibiotics and AMR	3	7	11	24	14	30	17	37	7	15	6
	I have sufficient knowledge about how to use antibiotics appropriately for my current practice	8	20	11	28	10	25	12	30	8	20	9
	I have a key role in helping control AMR	18	50	4	11	9	25	8	22	8	22	11
2 years	I know what antibiotic resistance is	1	1	2	2	4	4	25	22	81	72	0
	I know there is a connection between my prescribing/dispensing/administering	2	2	2	2	7	7	26	26	70	69	6

	of antibiotics and emergence and spread of resistant bacteria											
	I know what information to give to individuals about prudent use of antibiotics and AMR	2	2	16	16	23	22	39	38	28	27	5
	I have sufficient knowledge about how to use antibiotics appropriately for my current practice	4	5	17	22	24	30	30	38	21	27	17
	I have a key role in helping control AMR	20	29	17	25	17	25	16	23	21	30	22
3 years	I know what antibiotic resistance is	4	4	0	0	1	1	18	17	84	79	0
	I know there is a connection between my prescribing/dispensing/administering of antibiotics and emergence and spread of resistant bacteria	4	4	1	1	1	1	17	18	79	81	5
	I know what information to give to individuals about prudent use of antibiotics and AMR	3	3	5	5	24	23	47	45	27	26	1
	I have sufficient knowledge about how to use antibiotics appropriately for my current practice	1	1	7	8	23	26	48	54	19	21	9
	I have a key role in helping control AMR	11	12	24	25	17	18	22	23	21	22	0
4 years	I know what antibiotic resistance is	3	1	0	0	2	1	38	18	168	80	0
	I know there is a connection between my prescribing/dispensing/administering of antibiotics and emergence and spread of resistant bacteria	2	1	1	1	6	3	35	18	160	81	7

	I know what information to give to individuals about prudent use of antibiotics and AMR	3	1	10	5	59	29	91	45	44	22	4
	I have sufficient knowledge about how to use antibiotics appropriately for my current practice	6	3	25	14	69	37	74	40	24	13	13
	I have a key role in helping control AMR	22	16	24	17	39	28	45	32	46	33	35
5 years or more	I know what antibiotic resistance is	1	0	1	0	0	0	44	17	208	82	0
	I know there is a connection between my prescribing/dispensing/administering of antibiotics and emergence and spread of resistant bacteria	2	1	1	0	3	1	28	11	218	87	2
	I know what information to give to individuals about prudent use of antibiotics and AMR	0	0	4	2	32	13	135	54	82	33	1
	I have sufficient knowledge about how to use antibiotics appropriately for my current practice	10	4	9	4	74	32	115	49	36	15	10
	I have a key role in helping control AMR	40	21	21	11	42	22	52	27	69	36	30

Supplementary Material 6. Respondents' awareness of antibiotic awareness campaigns European Antibiotic Awareness Day (EAAD) and World Antibiotic Awareness Week (WAAW) and how effective they believe EAAD and WAAW have been in raising awareness about prudent use of antibiotics and antibiotic resistance in their country

**Degree**	**Have you heard of EAAD?**						**Have you heard of WAAW?**					
	Yes		No		Unsure		Yes		No		Unsure	
	n	%	n	%	n	%	N	%	N	%	n	%
All	216	24	623	68	80	9	230	25	612	67	75	8
Dentistry	6	14	34	79	3	7	7	16	33	76	3	7
Medicine	74	20	284	75	21	6	75	20	283	75	20	5
Nursing	12	11	83	79	10	10	18	17	80	76	7	7
Pharmacy	67	44	78	51	9	6	70	46	76	50	8	5
Scientists	13	29	27	59	6	13	11	24	27	59	8	17
Others	1	6	10	63	5	31	5	31	8	68	3	19

**Degree**	**Effectiveness of EAAD**						**Effectiveness of WAAW**					
	Effective		Undecided		Ineffective		Effective		Undecided		Ineffective	
	n	%	N	%	n	%	n	%	n	%	n	%
All	56	26	108	51	49	23	57	27	115	54	43	20
Dentistry	2	33	3	50	1	17	1	17	4	67	1	17
Medicine	13	19	39	56	18	26	14	19	43	60	15	21
Nursing	3	18	10	59	4	24	5	29	8	47	4	24
Pharmacy	23	35	32	49	11	17	23	34	35	52	9	13
Scientists	3	30	4	40	3	30	2	20	5	50	3	30
Others	1	20	3	60	1	20	1	17	4	67	1	17

Supplementary Material 7. Number and percentage of respondents who wished to receive additional information on topics relating to AMR and prudent antibiotic use

		Resistance to antibiotics	How to use antibiotics	Medical conditions for which antibiotics are used	Prescription of antibiotics	Links between the health of humans, animals and the environment	None
Yes (n=894)	n	564	486	384	399	485	40
	%	63	54	43	45	54	4
Dentistry (n=43)	n	31	27	20	27	19	1
	%	72	63	47	63	44	2
Medicine (n=379)	n	212	209	138	197	199	13
	%	56	55	36	52	53	3
Nursing (n=105)	n	79	54	57	35	69	4
	%	75	51	54	33	66	4
Pharmacy (n=154)	n	107	89	76	74	92	4
	%	69	58	49	48	60	3
Scientist (n=46)	n	34	22	22	12	24	1
	%	74	48	48	26	52	2
Other (n=16)	n	10	9	10	6	10	1
	%	63	56	63	38	63	6

Supplementary Material 8. Number of respondents by year of study who reported receiving teaching on prudent antibiotic use or infection management or practical experience through an internship/placement

		**Year of study**						
		1	2	3	4	5+	Unknown	Total
Received teaching on prudent antibiotic use (n = 758)	No	36	45	20	60	51	4	216
	Unsure	3	11	8	9	6	1	38
	Yes	20	57	80	142	197	8	504
Received teaching on infection management (	No	37	45	22	54	42	2	202
	Unsure	6	11	7	7	3	1	35
	Yes	16	57	79	150	209	10	521
Practical experience (n =751)	No	30	39	17	40	10	3	138
	Yes	29	72	88	169	244	14	613

Supplementary Material 9. Respondents' views on which teaching methods are effective to improve knowledge on the topic of prudent antibiotic use

	Agree		Unsure		Disagree		Missing
	n	%	n	%	n	%	n
Vignettes	399	81.9	48	9.9	40	8.2	735
Clinical placement (infectious diseases)	336	77.6	49	11.3	48	11.1	887
Lecture	428	74.8	78	13.6	66	11.5	650
Small group teaching	320	74.1	50	11.6	62	14.4	790
Peer teaching	242	63.5	71	18.6	68	17.8	841
Clinical placement (microbiology)	225	62.5	64	17.8	71	19.7	862
Active learning assessments	255	60.4	70	16.6	97	23.0	800
E-learning	171	46.5	89	24.2	108	29.3	854
Role Play	126	40.5	65	20.9	120	38.6	911
